# Parasitism of terrestrial gastropods by medically-important nematodes in Brazil

**DOI:** 10.3389/fvets.2022.1023426

**Published:** 2022-11-17

**Authors:** Silvana C. Thiengo, Jucicleide Ramos-de-Souza, Guilherme M. Silva, Monica A. Fernandez, Elizangela F. Silva, Arielly K. P. Sousa, Paulo S. Rodrigues, Aline C. Mattos, Ruam A. F. Costa, Suzete R. Gomes

**Affiliations:** ^1^Laboratório de Referência Nacional para Esquistossomose - Malacologia, Instituto Oswaldo Cruz, Fundação Oswaldo Cruz, Rio de Janeiro, Brazil; ^2^Programa de Pós-Graduação em Biologia Parasitária, Instituto Oswaldo Cruz, Fundação Oswaldo Cruz, Rio de Janeiro, Brazil; ^3^Laboratório de Biologia e Parasitologia de Mamíferos Silvestres Reservatórios, Instituto Oswaldo Cruz, Fundação Oswaldo Cruz, Rio de Janeiro, Brazil; ^4^Departamento de Metodologia da Enfermagem, Escola de Enfermagem Anna Nery, Universidade Federal do Rio de Janeiro - UFRJ, Rio de Janeiro, Brazil; ^5^Programa de Pós-Graduação em Saúde-Pública e Meio Ambiente, Escola Nacional de Saúde Pública Sérgio Arouca, Fundação Oswaldo Cruz, Rio de Janeiro, Brazil

**Keywords:** synanthropic gastropods, *Achatina fulica*, intermediate hosts, eosinophilic meningitis, *Angiostrongylus cantonensis*

## Abstract

An ample variety of parasitic associations are found between mollusks and nematodes, in which the mollusks may act as intermediate, paratenic or definitive hosts. Some free-living nematodes, in particular those of the order Rhabditida, are also found frequently in terrestrial mollusks. The present study reviews the results of the parasitological testing on samples of terrestrial mollusks conducted at the Brazilian National Reference Laboratory for Schistosomiasis and Malacology between 2008 and 2021. The samples were supplied primarily by the public health authorities from the different regions of Brazil, but also by research institutions and general population. The mollusks were processed individually and the obtained larvae were identified from their morphology and, whenever necessary, by molecular analysis. A total of 1,919 service orders were registered during the period, including 19,758 mollusk specimens collected from 23 of the 26 Brazilian states, as well as the Federal District, totalizing 145 municipalities. There was a marked predominance of the synanthropic species that are widely distributed in Brazil—*Achatina fulica* (87.08%), *Bulimulus tenuissimus* (4.18%), *Bradybaena similaris* (2.06%), and *Sarasinula linguaeformis* (1.50%). Of the 16,750 terrestrial mollusks examined, nematodes were recorded in 1,308 service orders, with the predominance of the superfamily Metastrongyloidea, in 616 service orders. They included *Angiostrongylus cantonensis*, rat lungworm, which was found in 252 samples, and *Aelurostrongylus abstrusus* in 145 samples. Free-living nematodes were found in 952 samples, *Ancylostoma caninum* and *Cruzia tentaculata* (previously identified as *Strongyluris* sp.) in one and 275 samples, respectively, and other parasites in 210 samples (not identified). The results highlight the diversity of the associations between nematodes and terrestrial mollusks in Brazil, in particular invasive and synanthropic species, with emphasis on the giant African land snail, *Achatina fulica*. They demonstrate the prominent role of this species of mollusk in the transmission of medically-important nematodes, which affect the health of both humans and animals, in particular eosinophilic meningitis, which is caused by *Angiostrongylus cantonensis*. This reinforces the need for more studies, and justify the growing demand for information as well as parasitological diagnosis of this mollusk, given its wide distribution in Brazil and its impact as an urban pest.

## Introduction

Many different types of association have been observed between nematodes and mollusks, including, paratenic, pathogenic, and parasitological relationships, which have been the subject of a wide range of studies since the early twentieth Century (1–9). These associations include dozens of species of mollusk that act as the intermediate hosts of medically-important nematodes that threaten the health of both humans and domestic animals (3, 7, 9–14).

Most of the nematodes of the superfamily Metastrongyloidea (order Strongylida) use mollusks as intermediate hosts (5, 15). This superfamily includes a number of medically-important species that affect the health of humans and animals, in particular *Angiostrongylus cantonensis* (Chen, 1935), which is the etiological agent of Eosinophilic Meningitis (EM), an emergent zoonosis in Brazil (7, 16–19). This nematode, which is endemic to Southeast Asia and the adjacent Pacific islands, was first recorded in Brazil in 2007, when it was found parasitizing the giant African land snail *Achatina* (*Lissachatina*) *fulica* Bowdich, 1822, in an epidemiological investigation of the first official death caused by this zoonosis in Brazil, recorded in the town of Cariacica, in the southeastern state of Espírito Santo (20). In 2009, two new cases were reported to the municipalities of Olinda and Escada, in the northeastern state of Pernambuco. In the case from Olinda, the epidemiological investigation revealed the presence of *A. fulica* specimens infected by *A. cantonensis* with a high parasitic load in the area surrounding the patient's residence. In Escada, by contrast, only specimens of the freshwater snail *Pomacea lineata* (Spix, *in* Wagner, 1827) were found infected with the parasite, but with low parasitic load. This is the first and unique record of the transmission of *Angiostrongylus* by freshwater snail in Brazil (21). Approximately 40 cases of EM have now been recorded in northern, northeastern, and southeastern Brazil (17, 22), although the true number of cases is likely to be much higher, due to the lack of knowledge of this zoonosis among healthcare workers (7).

*Achatina fulica* has expanded rapidly through much of Brazil, where it is now a common urban and agricultural pest, in addition to transmitting parasites (9, 23, 24), which has stimulated research into the nematodes associated with terrestrial mollusks. The situation has also attracted interest from both the Brazilian Health Ministry and a number of research groups seeking to determine the distribution of *A. cantonensis* in the country and the epidemiology of the transmission of EM. This increased demand for the services of the Laboratory of Malacology of the Oswaldo Cruz Institute in Rio de Janeiro and its Brazilian National Reference Laboratory for Schistosomiasis and Malacology (LRNEM), which is supported by the Health Ministry, to advance the identification of the nematodes associated with terrestrial mollusks, in particular *A. cantonensis*.

A number of other nematodes have not only been found in association with *A. fulica*, but also with other species of terrestrial mollusk, including both slugs and snails (9, 18, 22, 25–27). *Aelurostrongylus abstrusus* (Railliet, 1898), which is associated with *A. fulica* and other terrestrial mollusks, is medically important for domestic animals, causing cardiorespiratory problems in felids and canids (1, 9, 12, 28). Free-living nematodes such as those of the genera *Caenorhabditis* and *Rhabditis*, which are not considered to represent a threat to the health of humans or domestic animals (although they have been associated with otitis in bovines), have also been found in association with these mollusks (18, 27).

In addition to *A. fulica*, a number of different mollusks have been found to be naturally infected by *A. cantonensis*, both in Brazil and other New World countries (18, 19, 25, 29–31). The congeneric species Angiostrongylus costaricensis Morera and Céspedes, 1971, has been recorded from the southern United States southward as far as northern Argentina, where it parasitizes both wild and synanthropic rodents, as well as a number of different terrestrial mollusks, in particular the slugs of the family Veronicellidae (13, 32–34). While humans are only accidental hosts, *A. costaricensis* can cause abdominal angiostrongyliasis, with around 100 cases being reported from the states of southern (Rio Grande do Sul, Paraná, and Santa Catarina) and southeastern Brazil (Minas Gerais, Espírito Santo, Rio de Janeiro, and São Paulo), as well as the Federal District (13).

The present study analyzes and synthesizes the results of the parasitological tests conducted by the LRNEM between 2008 and 2021 aiming to provide an overview of the nematode parasites found in terrestrial mollusks in Brazil, in particular those that affect the health of both humans and domestic animals.

## Materials and methods

We analyze the results from parasitological analysis of mollusks received between 2008 and 2021 in the LRNEM of the Oswaldo Cruz Institute, Oswaldo Cruz Foundation (IOC/Fiocruz), that is part of the Brazilian Ministry of Health. Each sample of mollusks received by the LRNEM is registered on a service order form, where all the information on its origin and source is included, generating a service number. So, these mollusks are taxonomically identified and parasitologically analyzed to investigate their infection by nematodes. All information is then included in a digital database and sent to the entity or person that requested the analysis, which include health authorities such as state and municipal health secretariats, the Central Laboratory (LACEN) of different states, research institutions, and members of the general population.

For specific identification, mollusks were fixed in 70% alcohol to analyze morphological and conchological characteristics, following specialized catalogs (35–40) and by comparisons with specimens deposited in the Mollusk Collection of the Oswaldo Cruz Institute (CMIOC). Whenever possible, voucher specimens were deposited in the CMIOC. In some cases, we performed taxonomic updates of the mollusk species, considering published works and reviews made in recent years. This was the case for *Sarasinula linguaeformis* and *Megalobulimus dryades* (36, 41). The parasitological examination was based on the artificial digestion of the soft tissue in a 0.7% solution of hydrochloric acid, following the technique of Wallace and Rosen (3), as modified by Graeff-Teixeira and Morera (42), with each sample being analyzed individually (43).

However, it was not possible to identify the *Angiostrongylus* species reliably based on the morphological analysis of the larvae. Given this, the third stage larvae (L3) of the nematodes collected in 2008–2013, which were identified morphologically as metastrongyloids, were used to infect rodents experimentally, in order to obtain the adult form of the parasite for the analysis of its diagnostic morphological traits, which are missing in the larval form.

The growing demand for the identification of the nematode larvae found in the mollusk specimens, together with the limitations of these morphological identification methods, led to the implementation of molecular diagnostic techniques at the LRNEM, in order to identify the species more efficiently and rapidly. The molecular diagnosis of the metastrongyloid larvae by DNA sequencing was first adopted in 2013, with the procedures being adjusted gradually through the testing of different methods of DNA extraction, including both manual procedures and commercial kits. Between 2013 and 2021, then, partial CO1 and ITS2 sequences were obtained from the total DNA extracted from the larvae using the primers described by Folmer et al. (44), Prosser et al. (45), Bowles et al. (46), and Qvarnstrom et al. (47, 48). The objective of the Brazilian Ministry of Health is to know the places of positivity, regardless of the prevalence, as epidemiological surveillance actions are taken based mainly on the presence of the vector and/or the parasite in a given site. Therefore, LRNEM performed molecular analysis of a sample of Metastrongyloidea larvae from positive mollusks from the same sample/locality, but not for all of them.

The nematode *Ancylostoma caninum* was identified based on sequences of CO1 while *Aelurostrongylus abstrusus* and *Cruzia tentaculata* were usually identified morphologically, although the identification has been confirmed using COI and ITS2 sequences in some cases.

Amplified DNA fragments were purified and sent to the DNA Sequencing Platform (RPT01A) of the Fiocruz Technological Platforms Network, where the DNA was sequenced using the Sanger method, with the sequences then being edited in SeqMan. To confirm the identification of the species, the sequences were compared with those of known species available online to determine their similarity, using the BLAST tool available at https://blast.ncbi.nlm.nih.gov/Blast.cgi.

The sequences obtained from *A. cantonensis* were deposited in the LRNEM database, where they are available for multidisciplinary studies. Some of these sequences have been published previously and are available in GenBank, under Accession Numbers MH511539–MH511541 and MN994436-MN994438 (18, 22).

## Results

A total of 1,919 service orders were received in the LRNEM and analyzed in the present study, which included the results of the parasitological analyses and identification of terrestrial mollusks performed between 2008 and 2021. Overall, 19,758 specimens of snails and slugs were received from all the different Brazilian regions, including 23 states (145 municipalities), in addition to the Federal District (Brasília). Of this total of specimens received at the LRNEM, 16,750 were examined for infection by nematodes, given that many specimens were already dead when they arrived at the LRNEM or died before the parasitological examination, and some were fixed for the identification of species and deposited in the CMIOC.

**Figure 1:**
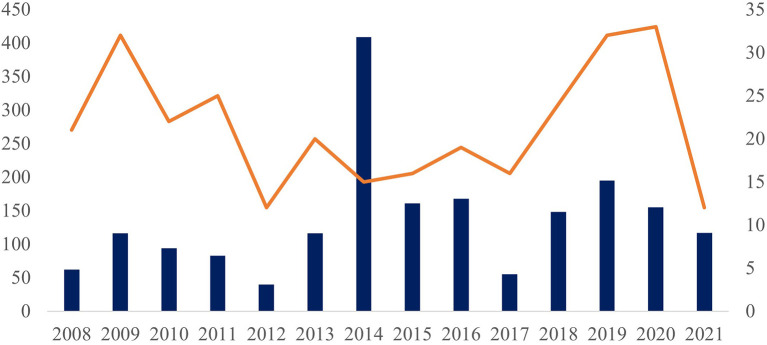
Number of test protocols (left side and columns) and number of municipalities of origin (right side and orange line) of the samples analyzed in the LRNEM between 2008 and 2021.

The largest number of service orders were received from the Southeast and Northeast regions, from where were received mollusks collected from 52 municipalities of the four southeastern Brazilian states (Espírito Santo, Minas Gerais, Rio de Janeiro, and São Paulo) and from 49 municipalities in eight northeastern states (Alagoas, Bahia, Ceará, Maranhão, Pernambuco, Piauí, Rio Grande do Norte, and Sergipe). Other sources included 12 municipalities from the Brazilian MidWest (Federal District and states of Goiás, Mato Grosso do Sul, and Mato Grosso), 16 municipalities in six northern states (Acre, Amazonas, Amapá, Pará, Roraima, and Tocantins), and 16 municipalities in two southern states (Santa Catarina and Paraná) (Supplementary Table 1). The number of service orders peaked in 2014, when 409 were registered at the LRNEM, based on samples received from 15 municipalities (Figure 1), which may reflect a concentration of surveys in a given period or locality. This information does not represent the mollusk population in these municipalities, but rather, it represents the interest of authorities in the identification and parasitological diagnosis of mollusks.

In fact, more than 200 of the samples received by the LRNEM in 2014 were collected in a single municipality of the state of Rio de Janeiro. This same municipality sent samples to the LRNEM every year between 2010 and 2020, including specimens collected from a number of different localities. Two states contributed the largest numbers of service orders—Rio de Janeiro, with specimens being collected in 37 municipalities, and Sergipe, from 21 municipalities.

**Figure 2:**
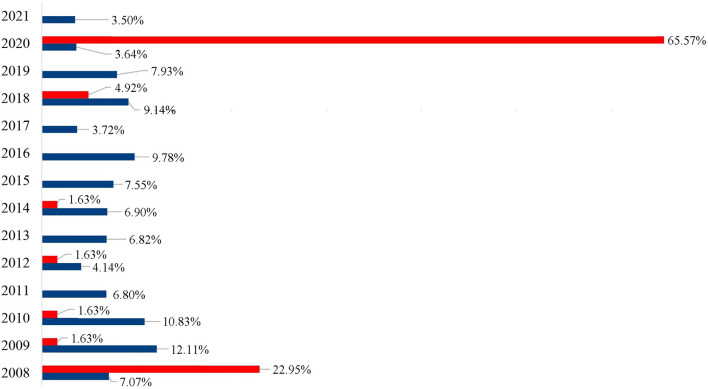
Percentage of the specimens of *Achatina fulica* (blue) and *Megalobulimus* sp. (red) registered per year by the LRNEM Reference Service between 2008 and 2021.

All mollusks received at the LRNEM and identified at species level are presented in Table 1. The four most frequently received species were *A. fulica, Bradybaena similaris, Bulimulus tenuissimus* and *S. linguaeformis*, which are all synanthropic species in Brazil (Table 1). *Achatina fulica* was by far the mollusk species with the largest number of specimens received at the LRNEM (*n* = 17,206; 87.08%). The number of specimens received of this last species by the LRNEM peaked in 2009 (Figure 2), when 2,085 specimens were received by the laboratory (12.12% of the total number of *A. fulica* specimens registered at the LRNEM).

**Table 1:** Taxa received and identified in specific level and their percentage in relation to the total of mollusks received at the LRNEM between 2008 and 2021, including those identified in genera level.

**Species**	***n***	**%**
*Achatina fulica*	17,206	87.08
*Bulimulus tenuissimus*	826	4.180
*Bradybaena similaris*	408	2.060
*Sarasinula linguaeformis*	297	1.500
*Leptinaria unilamellata*	229	1.160
*Subulina octona*	214	1.080
*Cyclodontina fasciata*	86	0.430
*Megalobulimus dryades*	24	0.120
*Macrochlamys indica*	27	1.360
*Diplosolenodes occidentalis*	20	0.100
*Megalobulimus ovatus*	16	0.080
*Succinea meridionalis*	14	0.070
*Ovachlamys fulgens*	10	0.050
*Allopeas gracile*	8	0.040
*Deroceras laeve*	8	0.040
*Limax flavus*	8	0.040
*Allopeas micra*	7	0.040
*Meghimatium pictum*	5	0.020
*Streptartemon cookeanus*	4	0.020
*Streptartemon deformis*	3	0.015
*Beckianum beckianum*	3	0.015
*Solaropsis brasiliensis*	2	0.010
*Solaropsis rosarium*	1	0.005
*Latipes erinaceus*	1	0.005
*Drymaeus papyraceus*	1	0.005

Some specimens, however, could not be identified to species level, including species of the genus *Bulimulus* (*n* = 93) specimens; 0.47% of the total registered by the LRNEM, *Megalobulimus* (21; 0.11%), *Omalonyx* (77; 0.39%), *Orthalicus* (07; 0.04%), *Rhinus* (01; 0.005%), *Sarasinula* (82; 0.51%), *Succinea* (02; 0.01%), and *Thaumastus* (27; 0.14%). Besides these, two specimens of the family Streptaxidae (0.01%) from Espírito Santo state.

It is also important to note that the LRNEM received many specimens of the native genus *Megalobulimus* that has been confused with *A. fulica* because of this large size and shell coloration. This genus was represented by 61 of the specimens received by the LRNEM, including *M. dryades* (24 specimens), *M. ovatus* (16 specimens), and *Megalobulimus* sp. (21 specimens). Almost two thirds (65.57%) of the *Megalobulimus* specimens were received in 2020, when 40 samples were registered. Whereas, specimens of *A. fulica* were registered at the LRNEM in all the years covered by the present study, *Megalobulimus* specimens were received in 7 years (Figure 2). In 2008, 1,217 *A. fulica* specimens were registered at the LRNEM (7.07%) of the total number of specimens of this species, followed by 2,085 in 2009 (12.12%), 1,864 in 2010 (10.83%), 1,171 in 2011 (6.81%), 714 in 2012 (4.15%), 1,175 in 2013 (6.83%), 1,188 in 2014 (6.9%), 1,300 in 2015 (7.56%), 1,684 in 2016 (9.79%), 641 in 2017 (3.73%), 1,574 in 2018 (9.15%), 1,364 in 2019 (7.93%), 626 in 2020 (3.64%), and 603 in 2021 (3.5%). In addition to the 40 *Megalobulimus* specimens received by the LRNEM in 2020, 14 specimens were received in 2008 (22.95% of the total), three in 2018 (4.92%), and just one each (representing 1.64% of the total in each case) in 2009, 2010, 2012, and 2014.

Considering the specimens examined parasitologically (not the number of received specimens), we observed that the vast majority (88.88%) of the 16,750 specimens examined parasitologically for the detection of nematodes were identified as *A. fulica* (*n* = 14,887 specimens), followed by *B. tenuissimus, B. similaris*, and *S. linguaeformis*, respectively. Some species were not examined parasitologically (*Beckianum beckianum, Ovachlamys fulgens, Rhinus* sp., *Solaropsis brasiliensis, Solaropsis rosarium, Streptartemon cookeanus, Streptartemon deformis*, and *Succinea meridionalis*), because they were used for the morphological identification and deposited subsequently in the CMIOC.

**Figure 3:**
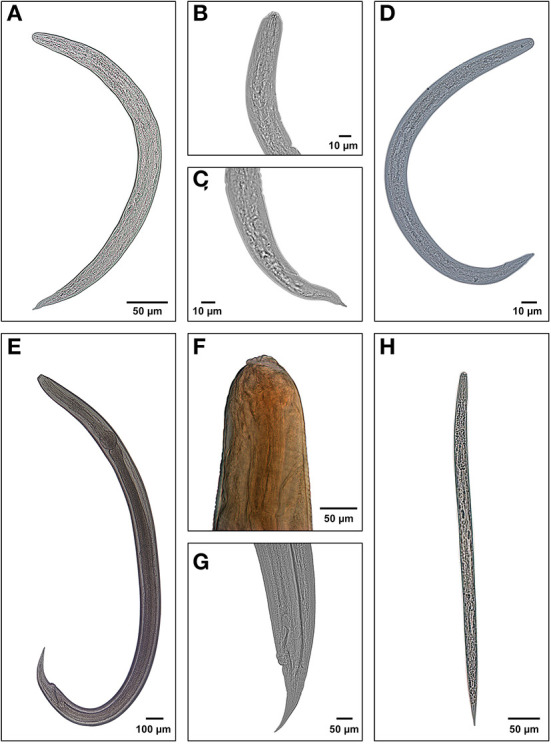
Larval forms of the nematodes recuperated from the mollusks examined at the LRNEM between 2008 and 2021: **(A)** whole L3 larva of *Angiostrongylus cantonensis*; **(B)** anterior extremity of *Aelurostrongylus abstrusus*; **(C)** posterior extremity of body; **(D)** whole L3 larva of *Aelurostrongylus abstrusus*; **(E)** whole larva of *Cruzia tentaculata*; **(F)** anterior extremity of *C. tentaculata*, showing the excretory pore; **(G)** posterior extremity of *C. tentaculata*, and **(H)** free-living nematode rabditiform.

The parasitological analysis showed larval forms of nematodes *Angiostrongylus cantonensis, Aelurostrongylus abstrusus* (both parasites of the superfamily Metastrongyloidea, family Angiostrongylidae), *Cruzia tentaculata* (Cosmocercoidea: Kathlaniidae) and *Ancylostoma caninum* (Ercolani, 1859) (Ancylostomatoidea: Ancylostomatidae) (Figure 3). In addition to these, free-living nematodes forms were observed in 952 samples (46.34% of the positive tests) and other identifications were not possible (210 samples, 10.22%). Metastrongyloidea larvae were identified in 616 samples (29.99%), *C. tentaculata* in 276 samples (13.44%) and *A. caninum* in one sample.

**Figure 4:**
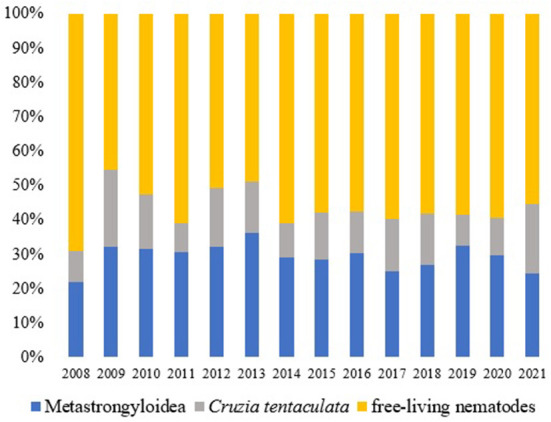
Nematodes (%) observed in the terrestrial mollusks examined at the LRNEM between 2008 and 2021.

During the analyzed period, there was a constancy in the records of nematodes (Figure 4).

*Angiostrongylus cantonensis* was recovered of 252 examined service orders. Of these 240 were obtained from samples of *Achatina fulica* (95.24%). In total, *A. cantonensis* was recovered from five species and one genus. The other species, included *S. linguaeformis* (7.41%), *B. similaris* (8.82%), *Subulina octona* (11.11%), *Sarasinula* sp. (5.00%), and *Cyclodontina fasciata* (14.29%) (Table 2). The parasitological analyses of *A. fulica* positive for *A. cantonensis* represent 15.51% of the total number of samples analyzed in this species (*n* = 240/1,547).

**Table 2:** Species found naturally infected with *Angiostrongylus cantonensis* from 2008 to 2021, followed by the number of analyzed service orders for these species (not considering 2,319 specimens received dead or fixed), number of infected service orders, and number of analyzed specimens in these service orders.

**Natural hosts**	**Analyzed service** **orders (*n*)**	**Service orders with** **infected samples (*n*)**	**Analyzed** **specimens (*n*)**
*Achatina fulica*	1,547	240	14,887
*Bradybaena similaris*	34	3	365
*Cyclodontina fasciata*	7	1	60
*Sarasinula linguaeformis*	54	4	219
*Sarasinula* sp.	20	1	82
*Subulina octona*	27	3	156
Total	1,689	252	15,769

LRNEM detects the nematodes of medical interest in a set of mollusks from a specific site (that here is represented by each service order).

**Figure 5:**
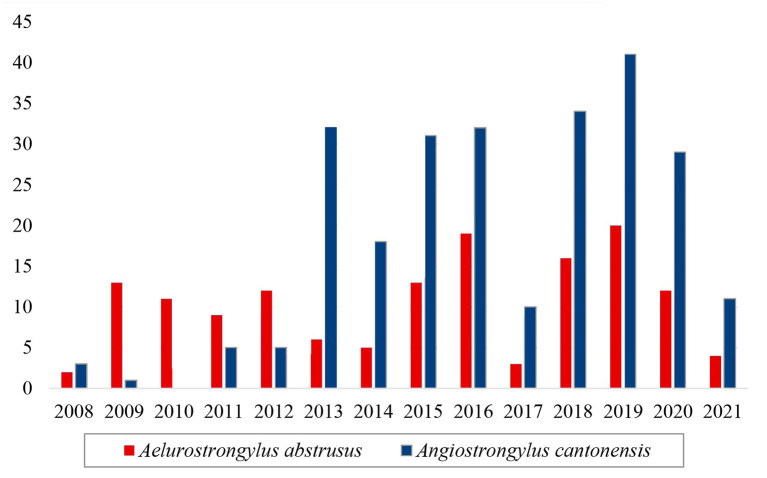
Samples of the family Angiostrongylidae (Metastrongyloidea) identified as *Aelurostrongylus abstrusus* (red) and *Angiostrongylus cantonensis* (blue) in the LRNEM between 2008 and 2021.

Considering another Metastrongyloidea, *Aelurostrongylus abstrusus* was identified in 145 service orders, and it was recorded in all the years of the study period (Figure 5). There was a notable increase in the number of positive tests for *Angiostrongylus cantonensis* after 2013 in comparison with the preceding years (Figure 5). This increase coincides with the implementation of the molecular diagnosis of the samples at the LRNEM.

**Figure 6:**
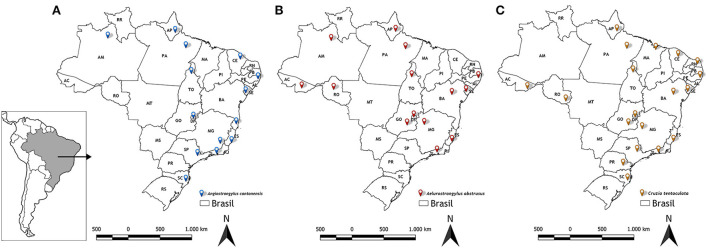
Distribution of nematodes obtained from LRNEM analyzes from 2008 to 2021. **(A)** *Angiostrongylus cantonensis*, **(B)** *Aelurostrongylus abstrusus*, and **(C)** *Cruzia tentaculata*.

Figure 6 shows the distribution of the three main nematode species of veterinary medical interest in Brazil carried out by terrestrial gastropods (*Angiostrongylus cantonensis, Aelurostrongylus abstrusus* and *Cruzia tentaculata*), according to the results obtained in the analyzes.

## Discussion

The records of the parasitological analyses of terrestrial mollusks conducted in the LRNEM over the past 14 years revealed the epidemiological importance of the synanthropic species, in particular *A. fulica*, as the intermediate hosts of zoonotic nematodes. Overall, the LRNEM complied with all requests made by the health authorities of 23 of the 26 Brazilian states, in addition to the Federal District. In many cases, infestation is excessive in Brazil, with *A. fulica* forming dense populations in urban areas. Previous records (23, 24) pointed that the most widespread infestations were recorded in the Brazilian states of Goiás (in 75 municipalities), São Paulo (69), Paraná (66), Rio de Janeiro (57), Mato Grosso (38), Espírito Santo (23), and Minas Gerais (20). In Brazil, the only state in which an infestation has not been recorded is Rio Grande do Sul, although even in this case, a juvenile specimen was collected recently in the municipality of Porto Alegre (49). *Achatina fulica* is also one of the world's worst invasive species, and is the most widespread exotic species in South America, where it is now found in 63 (58%) terrestrial ecoregions, 40 years after its initial introduction, in the 1980s (50).

Most of the service orders included mollusk samples collected in the Brazilian state of Rio de Janeiro, which may reflect the rapid expansion of *A. fulica* in this state (51), as well as the fact that the LRNEM is located in the state capital, Rio de Janeiro. Zanol et al. (51) recorded *A. fulica* in 26 of the 35 municipalities of Rio de Janeiro state considered by Thiengo et al. (23) to be free of infestation in 2006, what reflected the alarming growth in the number of municipalities infested in the state, over a period of only 4 years. *Achatina fulica* is now known to be present in all 26 Brazilian states (49).

We highlight the high number of specimens of *Megalobulimus* spp. received at the LRNEM. This genus includes the biggest native terrestrial that because of their big size and shell color have been confused to *A. fulica*. Also, *Megalobulimus* spp. also have been used in religious rituals and sold in local fairs in Brazil. The higher number of specimens received in 2020 were the result of specimens apprehended in a fair, where they were being illegally sold probably for this purpose.

*Angiostrongylus cantonensis* was identified most frequently in the service orders from different intermediate and definitive hosts collected from urban areas in northern, northeastern, southern, and southeastern Brazil (7) (Figure 6A). Overall, 240 of the positive tests of *Angiostrongylus cantonensis* referred to samples of *A. fulica* (Table 2), although this nematode was also identified in other terrestrial mollusks, i.e., *Sarasinula linguaeformis, Sarasinula* sp. *Bradybaena similaris, Subulina octona*, and *Cyclodontina fasciata*. The latter species is a native synanthropic snail from northeastern Brazil, which was identified only recently as a natural host of *Angiostrongylus cantonensis* (18). The other species are also synanthropic, and are amply distributed in Brazil (52).

*Angiostrongylus cantonensis* is known to be relatively unspecific in terms of its hosts (3, 25, 53). Valente et al. (19) also recorded other *Angiostrongylus* species in association with an ample variety of terrestrial mollusks, and identified *A. cantonensis* in 28 gastropod species, including 16 terrestrial snails, one freshwater snail (of the genus *Pomacea*), and 11 terrestrial slugs. Even so, *A. fulica* is by far the most prominent host, with the most widespread distribution of cases of *Angiostrongylus* infection in the Americas. Similarly, Graeff-Teixeira et al. (13) and Rambo et al. (32) reported that *Angiostrongylus costaricensis* has a diversity of mollusk hosts in southern Brazil, where abdominal angiostrongylosis is endemic, in particular, the native Brazilian slugs of the family Veronicellidae, as well as exotic slugs of the family Limacidae, and some snails (34).

Up to now, however, natural infection by *A. costaricensis* has not been recorded in *A. fulica*. Neuhauss et al. (54) recorded a low susceptibility of this mollusk to infection by the nematode under laboratory conditions. This may account for the lack of records of *A. costaricensis* in the records from the LRNEM, given that the vast majority of the samples are specimens of *A. fulica*.

*Aelurostrongylus abstrusus* is a cardiopulmonar parasite of felids, and was the nematode of veterinary concern found most frequently in the mollusks examined at the LRNEM. Cases of infection by this nematode have been increasing in recent years in many parts of the world, including Brazil, where it has attracted increasing attention from veterinarians (1, 9, 12, 55).

Like *Angiostrongylus cantonensis, Aelurostrongylus abstrusus* has an indirect life cycle, in which the L_1_ larvae are released into the faces of the felids, which are the definitive hosts. Rats and birds that ingest infected snails may act as paratenic hosts. The cats are infected by ingesting parasitized snails or paratenic hosts (28). However, few studies have identified which mollusk species act as intermediate hosts of this nematode. Lima et al. (55) reviewed the data on the occurrence of this nematode species in Brazil, and found that *A. fulica* was the only species recognized as an intermediate host of *A. abstrusus* anywhere in the country. In particular, in a parasitological study of terrestrial mollusks collected from 46 municipalities of the Metropolitan and Central Fluminense mesoregions of the state do Rio de Janeiro, Rodrigues et al. (9) found that 99% of the mollusks infected with *A. abstrusus* were specimens of *A. fulica*, although they did also record infection in a native slug, *Latipes erinaceus* (Colosi, 1921), from an area in which *A. fulica* was not found. The results of the present study also emphasize the role of *A. fulica* as an intermediate host of this nematode (Figure 6B). While *A. abstrusus* was recorded infecting other four mollusk species (*Allopeas gracile, Bradybaena similaris, L. erinaceus* and *Thaumastus* sp.), by far the largest number of specimens infected were of *A. fulica*, although this was obviously determined, in part, by the much larger numbers of this snail sent to the LRNEM.

Free-living nematodes, such as those of the genera *Caenorhabditis* and *Rhabditis*, which have been associated with the occurrence of otitis in bovines (56), have also been found in *A. fulica*, in both the adult and larval stages (18, 27). These nematodes may exploit *A. fulica* as a phoretic host, by attaching themselves to the external mucus of the snail or by passing through its digestive system (57). In Brazil, free-living nematodes are represented by an enormous diversity of species, including rhabiditform nematodes in different genera (56, 58). The nematodes of the genus *Rhabditis* are found typically in decomposing organic matter and humid soil (27). *Achatina fulica* is generally associated with marginal urban environments that have accumulations of waste and rubble, a lack of basic sanitation, and decomposing organic matter (27, 59). These observations are consistent with the results of the present study, given that the majority of the positive reports emitted by the LRNEM (*n* = 952; 72.78%) indicate an association between *A. fulica* and free-living nematodes.

In the case of the larvae that had previously been identified as *Strongyluris* sp., a recent study at the LRNEM (26), based on an integrated morpho-molecular analysis of the specimens, concluded that the samples identified as *Strongyluris* sp. did in fact represent *Cruzia tentaculata* (Rud, 1819) (Figures 3E–G), a parasite of the cecum of opossums (*Didelphis* spp.) (60–62). Ever since it was described, the life cycle of *C. tentaculata* has been considered monoxenic (62, 63), due to the fact that the intermediate host of its larval phase was unknown. Larvae identified morphologically as *Strongyluris* sp. or *Strongyluris*-like have been reported in mollusks since the 1990s (18, 64–66). However, including reports from the LRNEM, it is clear that this nematode is found in a number of different species of terrestrial slugs and snails.

The strongylid nematode reported here, *Ancylostoma caninum*, was identified in a single sample of *Achatina fulica* received in 2015 from the southeastern Brazilian state of Minas Gerais. This nematode, which has free-living larvae, is also a medically-important helminth, given that canids are its principal definitive hosts. It causes chronic gastroenteritis, with blood loss that evolves to anemia, in addition to infecting humans accidentally. In humans, it causes a cutaneous infection (larva migrans) and, more rarely, eosinophilic enteritis (67, 68). While this type of infection has been reported previously in mollusks, including *A. fulica* (68), this is the first record of *A. caninum* infecting *Achatina fulica* in Brazil. This interspecific interaction, which was reported for the first time in 2014, in the Philippines, indicates the possibility of a third route of infection of the larvae of this nematode to the definitive hosts (canids), as reported by Constantino-Santos et al. (68). This nematode has also been observed infecting marsupials of the genus *Didelphis* (opossums), which often feed on mollusks (69). While this is probably an accidental interaction, rather than an integral component of the life cycle of *A. caninum*, these records emphasize the acting of *A. fulica* as a disseminator of an ample variety of zoonotic helminths albeit accidentally.

The interactions between mollusks and nematodes reported in the present study reinforce the need for further, similar studies that will enhance the interpretation of this association. This will be important not only for understanding of the epidemiology of transmission, but also the development of measures to prevent the transmission and control of the parasitosis carried out by terrestrial mollusks in Brazil, especially in urban areas where synanthropic and exotic mollusks are present.

## Data availability statement

The datasets presented in this study can be found in online repositories as genetic data are published, such as those cited in the text.

## Author contributions

ST, JR-d-S, SG, and MF wrote and reviewed the text and database. JR-d-S, GS, ES, AS, PR, AM, and RC tabulated the database. All authors participated in the writing of the text and approved the submitted version.

## Funding

This work was supported by Instituto Oswaldo Cruz/Fiocruz and Ministry of Health.

## Conflict of interest

The authors declare that the research was conducted in the absence of any commercial or financial relationships that could be construed as a potential conflict of interest.

## Publisher's note

All claims expressed in this article are solely those of the authors and do not necessarily represent those of their affiliated organizations, or those of the publisher, the editors and the reviewers. Any product that may be evaluated in this article, or claim that may be made by its manufacturer, is not guaranteed or endorsed by the publisher.

## Supplementary material

Supplementary Table 1 Number of positive orders for nematodes and total of analyzed specimens received at the LRNEM between 2008 and 2021, indicating their hosts, number of municipalities by State and respective Brazilian Region.
