# Supplementary material for: Parasitism of terrestrial gastropods by medically-important nematodes in Brazil
Source: Front Vet Sci. 2022 Nov 17;9:1023426. doi: 10.3389/fvets.2022.1023426 (PMC9715018; doi:10.3389/fvets.2022.1023426)
Supplement: Supplementary Table 1 — Number of positive orders for nematodes and total of analyzed specimens received at the LRNEM between 2008 and 2021, indicating their hosts, number of municipalities by State and respective Brazilian Region. [file Table_1.docx]

**Supplementary file Table 1**. Number of positive orders for nematodes and total of analyzed specimens received at the LRNEM between 2008 and 2021, indicating their hosts, number of municipalities by State and respective Brazilian Region.

| **Região** | **State** | **Municipality (n)** | **Hosts** | **Analyzed specimens (n)** | **Positive service orders (n)** |
| --- | --- | --- | --- | --- | --- |
| **Midwest** | | |  |  |  |
|  | DF | 01 | *Achatina fulica* | 159 | 06 |
|  |  |  | *Bradybaena similaris* | 210 | 07 |
|  |  |  | *Bulimulus tenuissimus* | 09 | 00 |
|  |  |  | *Deroceras laeve* | 06 | 00 |
|  |  |  | *Leptinaria unilamellata* | 13 | 01 |
|  |  |  | *Sarasinula linguaeformis* | 21 | 00 |
|  |  |  | *Sarasinula* sp. | 03 | 00 |
|  |  |  | *Subulina octona* | 04 | 00 |
|  |  |  |  |  |  |
|  | GO | 08 | *Achatina fulica* | 390 | 14 |
|  |  |  | *Bradybaena similaris* | 01 | 00 |
|  |  |  | *Sarasinula* sp. | 05 | 01 |
|  |  |  |  |  |  |
|  | MS | 01 | *Achatina fulica* | 04 | 01 |
|  |  |  |  |  |  |
|  | MT | 01 | *Subulina octona* | 08 | 00 |
|  |  |  |  |  |  |
| **Northeast** | | |  |  |  |
|  | AL | 01 | *Achatina fulica* | 11 | 01 |
|  |  |  |  |  |  |
|  | BA | 06 | *Achatina fulica* | 287 | 12 |
|  |  |  | *Bulimulus tenuissimus* | 06 | 01 |
|  |  |  | *Subulina octona* | 26 | 00 |
|  |  |  |  |  |  |
|  | CE | 11 | *Achatina fulica* | 241 | 21 |
|  |  |  | *Bradybaena similaris* | 09 | 00 |
|  |  |  |  |  |  |
|  | MA | 02 | *Achatina fulica* | 259 | 08 |
|  |  |  |  |  |  |
|  | PE | 05 | *Achatina fulica* | 161 | 17 |
|  |  |  | *Leptinaria unilamellata* | 128 | 03 |
|  |  |  | *Sarasinula linguaeformis* | 24 | 03 |
|  |  |  | *Sarasinula* sp. | 11 | 02 |
|  |  |  | *Subulina octona* | 08 | 00 |
|  |  |  |  |  |  |
|  | PI | 01 | *Achatina fulica* | 22 | 1 |
|  |  |  |  |  |  |
|  | RN | 03 | *Achatina fulica* | 271 | 21 |
|  |  |  |  |  |  |
|  | SE | 20 | *Achatina fulica* | 500 | 70 |
|  |  |  | *Bulimulus tenuissimus* | 26 | 05 |
|  |  |  | *Cyclodontina fasciata* | 60 | 04 |
|  |  |  | *Leptinaria unilamellata* | 10 | 01 |
|  |  |  | *Sarasinula linguaeformis* | 17 | 04 |
|  |  |  | *Sarasinula* sp. | 06 | 02 |
|  |  |  | *Subulina octona* | 03 | 01 |
|  | |  |  |  |  |
| **North** | AC | 01 | *Achatina fulica* | 37 | 10 |
|  |  |  | *Allopeas gracile* | 04 | 01 |
|  |  |  |  |  |  |
|  | AM | 01 | *Achatina fulica* | 27 | 03 |
|  |  |  |  |  |  |
|  | AP | 01 | *Achatina fulica* | 193 | 40 |
|  |  |  | *Bulimulus tenuissimus* | 49 | 11 |
|  |  |  | *Deroceras laeve* | 19 | 01 |
|  |  |  | *Sarasinula linguaeformis* | 35 | 11 |
|  |  |  | *Sarasinula* sp. | 05 | 01 |
|  |  |  | *Subulina octona* | 26 | 03 |
|  |  |  |  |  |  |
|  | PA | 07 | *Achatina fulica* | 255 | 24 |
|  |  |  |  |  |  |
|  | RO | 02 | *Achatina fulica* | 158 | 05 |
|  |  |  |  |  |  |
|  | TO | 04 | *Achatina fulica* | 284 | 21 |
|  |  |  | *Bulimulus tenuissimus* | 02 | 01 |
|  |  |  | *Subulina octona* | 19 | 00 |
| **Southeast** | |  |  |  |  |
|  | ES | 04 | *Achatina fulica* | 203 | 19 |
|  |  |  | *Bradybaena similaris* | 07 | 02 |
|  |  |  | *Bulimulus tenuissimus* | 2 | 01 |
|  |  |  | *Sarasinula linguaeformis* | 26 | 01 |
|  |  |  | *Sarasinula* sp. | 04 | 01 |
|  |  |  | Streptaxidae | 02 | 00 |
|  |  |  | *Subulina octona* | 01 | 00 |
|  |  |  |  |  |  |
|  | MG | 06 | *Achatina fulica* | 282 | 20 |
|  |  |  | *Megalobulimus* sp. | 01 | 01 |
|  |  |  |  |  |  |
|  | RJ | 37 | *Achatina fulica* | 6407 | 657 |
|  |  |  | *Bradybaena similaris* | 92 | 06 |
|  |  |  | *Bulimulus tenuissimus* | 181 | 09 |
|  |  |  | *Leptinaria unilamellata* | 16 | 01 |
|  |  |  | *Omalonyx* sp. | 01 | 01 |
|  |  |  | *Sarasinula linguaeformis* | 96 | 08 |
|  |  |  | *Sarasinula* sp. | 48 | 02 |
|  |  |  | *Subulina octona* | 61 | 02 |
|  |  |  | *Thaumastus* sp. | 01 | 00 |
|  |  |  |  |  |  |
|  | SP | 05 | *Achatina fulica* | 771 | 44 |
|  |  |  | *Orthalicus* sp. | 03 | 00 |
| **South** | |  |  |  |  |
|  | PR | 07 | *Achatina fulica* | 322 | 16 |
|  |  |  | *Bulimulus tenuissimus* | 428 | 01 |
|  |  |  | *Limax flavus* | 07 | 00 |
|  |  |  | *Meghimatium pictum* | 05 | 00 |
|  |  |  |  |  |  |
|  | SC | 10 | *Achatina fulica* | 3643 | 167 |
|  |  |  | *Bradybaena similaris* | 46 | 01 |
